# Practice reduces task relevant variance modulation and forms nominal trajectory

**DOI:** 10.1038/srep17659

**Published:** 2015-12-07

**Authors:** Rieko Osu, Ken-ichi Morishige, Jun Nakanishi, Hiroyuki Miyamoto, Mitsuo Kawato

**Affiliations:** 1 ATR Brain Information Communication Research Laboratory Group, Keihanna Science City, Kyoto, Japan; 2 Department of Intelligent Systems Design Engineering, Toyama Prefectural University, Imizu, Toyama, Japan; 3 Institute for Cognitive Systems, Technical University of Munich, Munich, Germany; 4 Graduate School of Life Science and Systems Engineering, Kyushu Institute of Technology, Kitakyushu, Japan

## Abstract

Humans are capable of achieving complex tasks with redundant degrees of freedom. Much attention has been paid to task relevant variance modulation as an indication of online feedback control strategies to cope with motor variability. Meanwhile, it has been discussed that the brain learns internal models of environments to realize feedforward control with nominal trajectories. Here we examined trajectory variance in both spatial and temporal domains to elucidate the relative contribution of these control schemas. We asked subjects to learn reaching movements with multiple via-points, and found that hand trajectories converged to stereotyped trajectories with the reduction of task relevant variance modulation as learning proceeded. Furthermore, variance reduction was not always associated with task constraints but was highly correlated with the velocity profile. A model assuming noise both on the nominal trajectory and motor command was able to reproduce the observed variance modulation, supporting an expression of nominal trajectories in the brain. The learning-related decrease in task-relevant modulation revealed a reduction in the influence of optimal feedback around the task constraints. After practice, the major part of computation seems to be taken over by the feedforward controller around the nominal trajectory with feedback added only when it becomes necessary.

Biological motor control problems involve considerable redundancy, neural noise, and substantial sensorimotor delay. The brain solves these problems with limited resources and time by learning to perform given tasks. In particular, the brain has to cope with the variance caused by external perturbation as well as internally generated noise. Recent studies suggested that the variance was reduced only around the task constraints and remained large at locations irrelevant to the task in complex tasks, which is called the minimal intervention principle. At the same time, there is much experimental evidence showing a reduction of movement variance after learning and convergence to nominal trajectories in simple reaching tasks.

These two different observations of movement variance are closely related with old and new arguments of whether biological motor systems mainly depend on feedback control or feedforward control. In the optimal feedback control approach, the brain does not plan movement beforehand, but solves the problems online using all possible feedback information available at that time without the need for feedforward control^1^. This idea originated in traditional psychological theory such as dynamical system theory^2^,^3^ and the theory of uncontrolled manifolds^4^,^5^,^6^, and was formulated in the context of biological motor control as the optimal feedback control theory by Todorov and Jordan^1^. This approach solves all problems simultaneously at the feedback level and therefore assumes a heterarchical implementation in the brain. There is a large amount of behavioral evidence that humans reduce variability only in the direction relevant to the task^4^,^6^,^7^. Such task-relevant variance modulation is called the minimal intervention principle and has been regarded as evidence of the absence of a plan^1^,^8^,^9^. Feedforward control, on the other hand, solves the problem sequentially by dividing a complicated problem into several simple problems (divide and conquer). To divide the problem, this approach normally requires an intermediate representation between the task and motor commands, e.g., a desired trajectory, that are not directly specified by the task constraint. Such a strategy assumes hierarchical implementation in the brain. Complicated problems are partly solved at the planning level before the start of movement^10^,^11^,^12^,^13^,^14^. This type of approach stems from control theory in robotics, and physiological and imaging data suggest hierarchical information processing and modular characteristics of the brain^15^,^16^,^17^. If the role of feedback control (such as the control of impedance) is to reduce the deviation from the planned (desired) trajectory, the actual trajectories are likely to be spatially and temporally uniformly distributed around the fixed planned trajectory, rather than reducing their variance only around task constraints.

The question we address in this paper is whether task relevant modulation (optimal online feedback control) is the major contribution of the trajectory variance, or whether the assumption of nominal trajectories (feedforward control) is required for explaining trajectory variance. As a behavioral investigation, we focused on the modulation of variance in reaching tasks with multiple intermediate targets.

We first focus on the learning-related change of task-relevant modulation in variance. An important prediction to be tested is if the minimal intervention principle is optimal behavior in the sense of the optimal feedback control schema, then task-relevant modulation should be observed in skilled movements even after learning. Our behavioral experiments demonstrate that this task-relevant modulation decreases or disappears after practice, suggesting the significance of plan-based control for skilled movements.

We then examine the possibility that movement planning is expressed in the form of trajectories (i.e., kinematic variables as a function of time) in the brain. In such a case, we suppose that neural noise could be added to the time indices (time-jitter noise), which predicts velocity-dependent modulation of variability. The observed velocity-dependent modulation of the trajectory variability would support the existence of nominal trajectory expression in the brain with the proposed time-jitter noise model.

We finally consider the effect of dynamics of the body (e.g., arm dynamics) on the observed variance modulation. The experimental paradigm suggests that trajectory optimization should take into account the dynamics of the nonlinear musculoskeletal system, which makes the problem of online optimization complex.

## Results

### Experiment 1: task-relevant variance modulation disappears after learning

We asked participants to perform reaching tasks with multiple targets and examined the practice-related changes in the variance structure.

**Figure 1:**
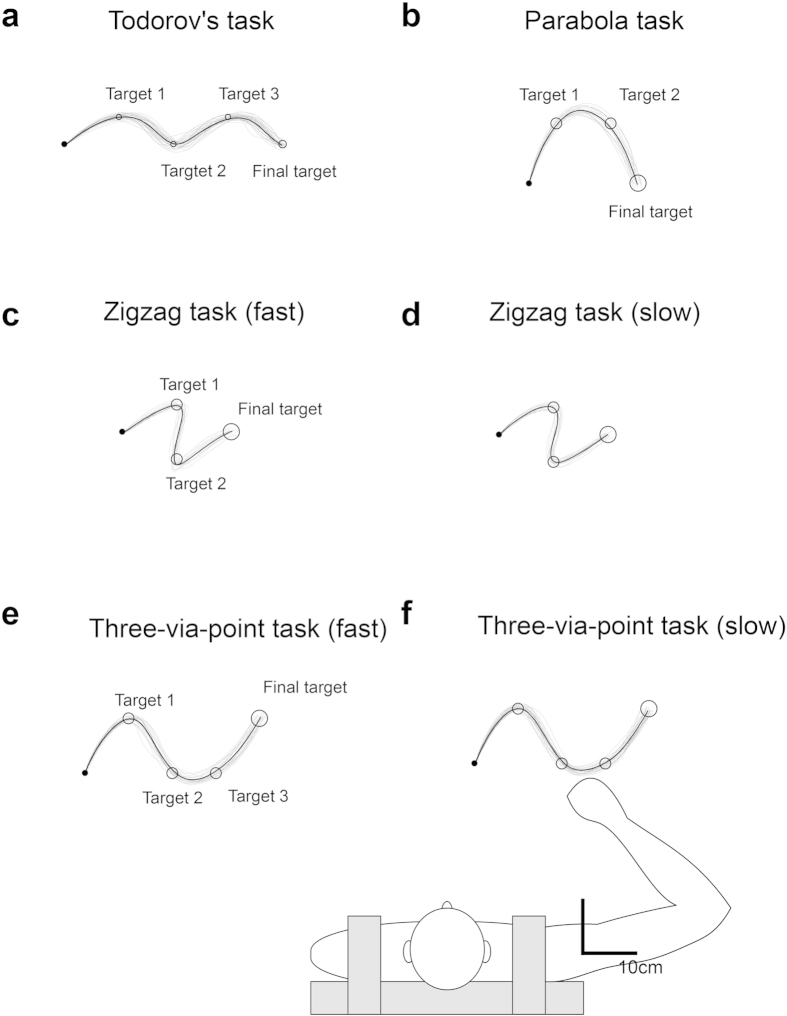
Allocations of targets and examples of hand paths for each task. In Todorov’s task, three intermediate targets were located in the same way as the multiple target condition of Experiment 1 in ref. 14; i.e., the first target was located distally, the second proximally, and the third distally. In the parabola task, two intermediate targets were located distally. In the zigzag task, the first target was located distally and the second proximally. In the three via-point task, the first target was located distally, with the second and the third located proximally. The participants were tasked with moving their hand from the start point (a circle with a radius of 0.5 cm) to the final target (a circle with a radius of 1.5 cm) by passing through intermediate targets (a circle with a radius of 1 cm) within a time limit (Todorov’s task: 1350 ± 150 ms; parabola task: 800 ± 50 ms; zigzag fast: 1000 ± 50 ms; zigzag slow: 1500 ± 75 ms; three-via-point fast: 1200 ± 50 ms; three-via-point slow: 1800 ± 75 ms). The filled circles denote the start circle and the open circles denote the target circles. The gray curves denote hand paths of participants after learning (last 20% of all trials). Black curves are average hand paths.

**Figure 2:**
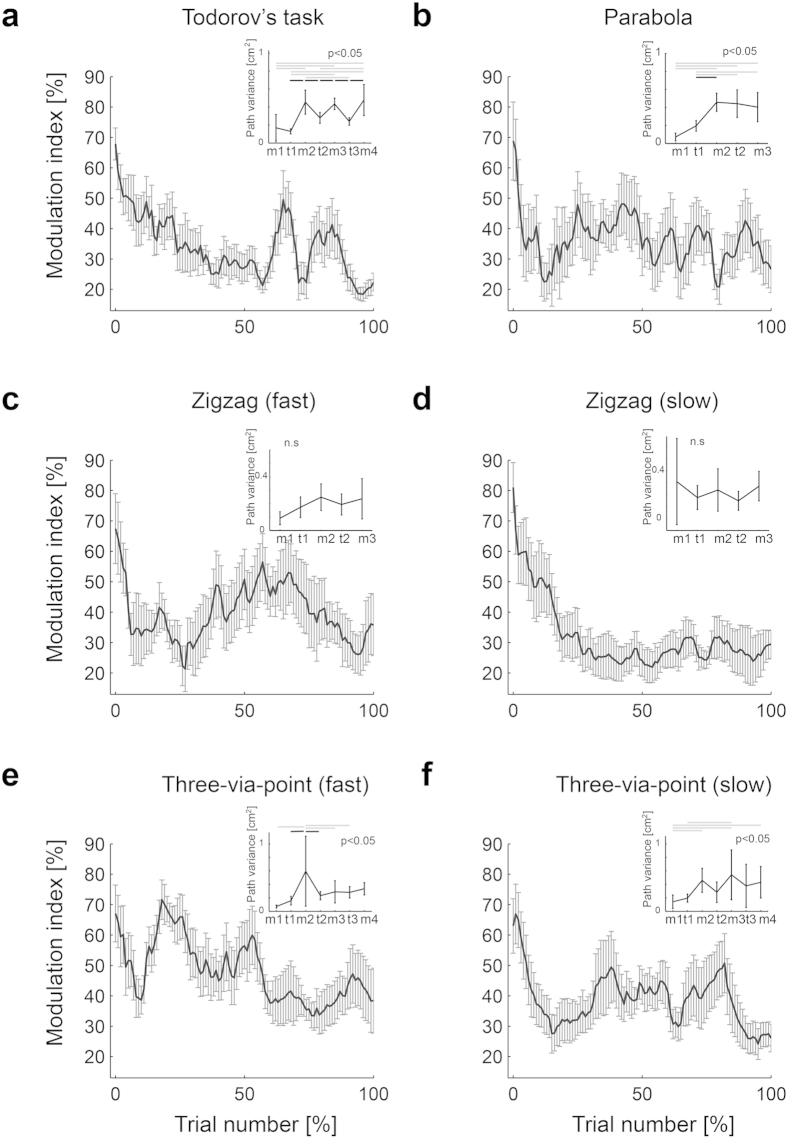
Learning-related decrease in variance modulation. The solid curves show the time course of modulation indices ensemble averaged across participants. The error bars denote their standard errors. The time course modulation indices for each participant are computed over the moving window of 15 trials and normalized by the maximum value. Trial numbers are normalized so that the total numbers are 100. Each inset shows the path variance at each intermediate target and midpoints between targets computed from the last 15 trials. In the inset, m1, m2, and m3 of the horizontal axis correspond to the midpoints between intermediate targets where the significant increase in the path variance relative to the adjacent targets is predicted according to the minimal intervention principle. t1, t2 and t3 correspond to the intermediate target positions where the significant decrease in the path variance is predicted according to the minimal intervention principle. The black horizontal bar denotes significant post-hoc comparisons observed between the adjacent target and midpoint that correspond to the minimal intervention principle (a significantly smaller variance in the target compared with the adjacent midpoint). Gray horizontal bars denote other cases. Significant modulation after learning corresponding to the minimum intervention principle was observed only in the early part of the fast multi-target task.

The participants performed four different types of multiple-target movements until 50 successful trials were acquired (see Fig. 1 and Methods). To focus on variance modulation in the task space, we computed the variance normalized by path length^1^ (see Eq. 2). This ‘path variance’ evaluates solely spatial variance by selecting the nearest points, without taking into account the temporal information^1^. To quantify the variance modulation, we computed the modulation index (MI) from path variance. The MI will be large if the variances between targets are larger than the variance at adjacent targets. Therefore, a larger MI indicates better modulation of the variance. Figure 2 shows the evolution of the MI over normalized trial numbers. In general, the task-relevant modulation of the path variance was observed at the early stage of practice, and reduced at the later stage of practice. In all tasks, the MI gradually decreased as practice proceeded. In five of six tasks, the MI of the final 5% of total trials was significantly smaller than that of the initial 5% (Todorov’s task: t(8) = 7.11, p = 0.00010, parabola task: t(7) = 2.83, p = 0.02544, fast zigzag task: t(7) = 2.12, p = 0.07137, slow zigzag task: t(8) = 5.20, p = 0.00082, fast three-via-point task: t(6) = 2.53, p = 0.04440, slow three-via-point task: t(8) = 3.14, p = 0.01383). The path variance at each target did not change significantly while it significantly decreased after learning (paired t-tests) at some midpoints between the targets. Therefore, the observed decrease in the modulation index was due not to the increase in variance at the targets but to the decrease in variance at the midpoints.

We then examined whether there was still significant modulation of path variance after learning (inset of Fig. 2). Although we observed significant differences among targets and midpoints in Todorov’s task, the parabola task, and the three-via-point tasks (ANOVA), the post-hoc comparison revealed that there were differences relevant to the minimum intervention principle (i.e., an increase in variance at a midpoint in comparison with its neighboring targets) for midpoints 2 and 3 of Todorov’s task and for midpoint 2 of the three-via-point task, but not for others. These results show that task-relevant modulation of path variance does not necessarily increase but tends to disappear after practice.

If online correction towards the original task constraints is the major contribution of the variance modulation, it is hard to explain why the variance between the targets has reduced, where no task constraints were given. Reduced task relevant modulation after learning suggests that the brain may have been lead to converge the trajectory between the targets through practice, by learning a nominal trajectory toward which the variance is reduced.

### Temporal aspect of variance modulation

**Figure 3:**
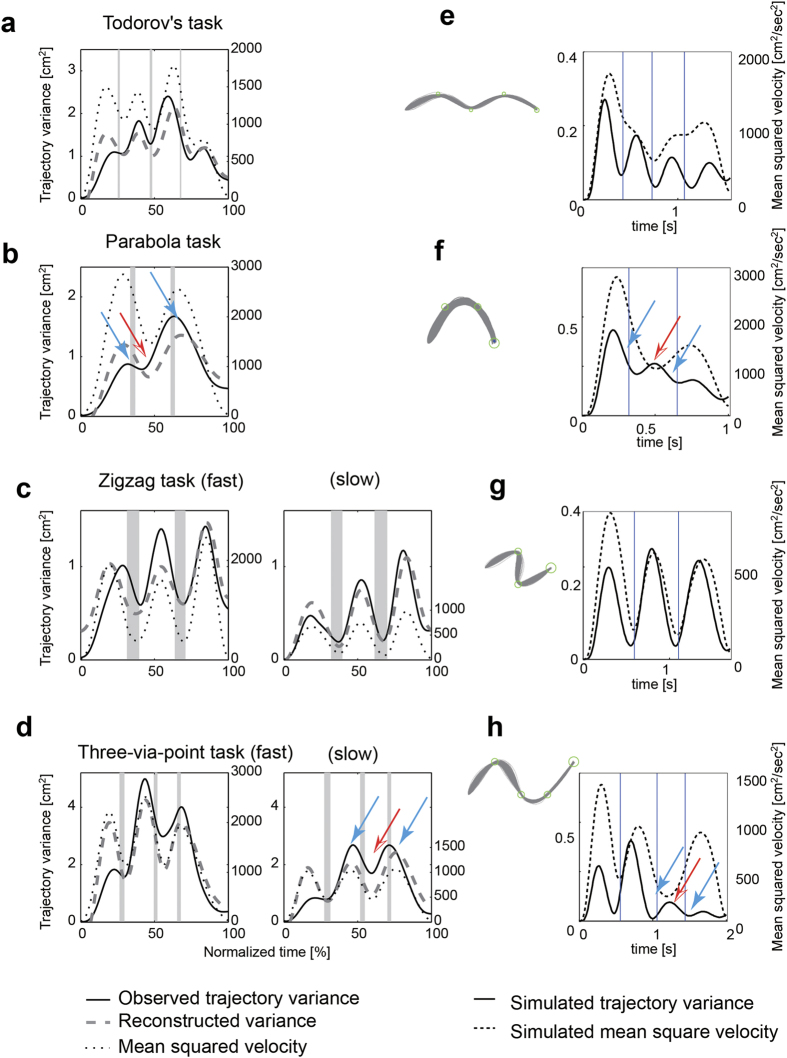
Velocity-dependent modulation of trajectory variance and OFC simulation. (**a–d**) Time course of trajectory variance that considers the temporal aspect of the variance for typical participants. Solid curves are observed trajectory variance. The black dotted curves show the mean squared tangential velocity. Gray dash curves demonstrate trajectory variance reconstructed from the mean squared tangential velocity and the signal-dependent noise term from Equation 1. Gray areas denote the duration that the hand stayed within via-point circles. (**e–h**) Results of LQG simulations. Left panels show hand paths. Right panels show time courses of trajectory variance and mean squared velocity. Vertical lines denote the time specified in the simulation when the hand passes through targets. The simulation parameters (*σ*_*S*_ = 0.4, *σ*_*u*_ = 0.7, *w*_*v*_ = 0.1, *w*_*f*_ = 0.01, r = 0.002) were set so that the path variance appears similar. The times when the hand passed through targets were obtained from the data.

We then computed the ‘trajectory variance’ taking into account the spatio-temporal characteristics of the variance. Here the trajectory variance is defined as the spatial variance along normalized time, and the effect of movement duration was removed by resampling the position for each trajectory so that the duration was evenly divided into 100 pieces (time normalization, as detailed in Methods). This method normalizes the total movement duration without removing the local time warp. While the path variance is expressed as a function of path length, the trajectory variance is expressed as a function of normalized time. Figure 3a–d show the temporal profiles of the trajectory variance and mean squared velocity for representative participants. In all tasks, there was a general tendency that the trajectory variance increased with an increase in velocity and vice versa. When the minima of the velocity and target did not match (e.g., targets 1 and 2 in the parabola task, targets 2 and 3 in the multi-target task), the minima of the trajectory variances were located not around the targets (blue arrows) but around the midpoints where the velocity minima were observed (red arrows). These results suggest that the trajectory variance did not follow the minimal intervention principle but changed in parallel with the velocity profile. As expected, the linear quadratic Gaussian (LQG) framework^1^,^18^ that computes optimal feedback controllers predicted the minimal intervention principle even for the parabola task and the multi-target task, in which the variance increased at the mid-point of the two via-points where the velocity was minimal (red arrows in Fig. 3f and h, in comparison with those in Fig. 3b,d).

### Experiment 2: noise in the nominal trajectory explains movement variance

**Figure 4:**
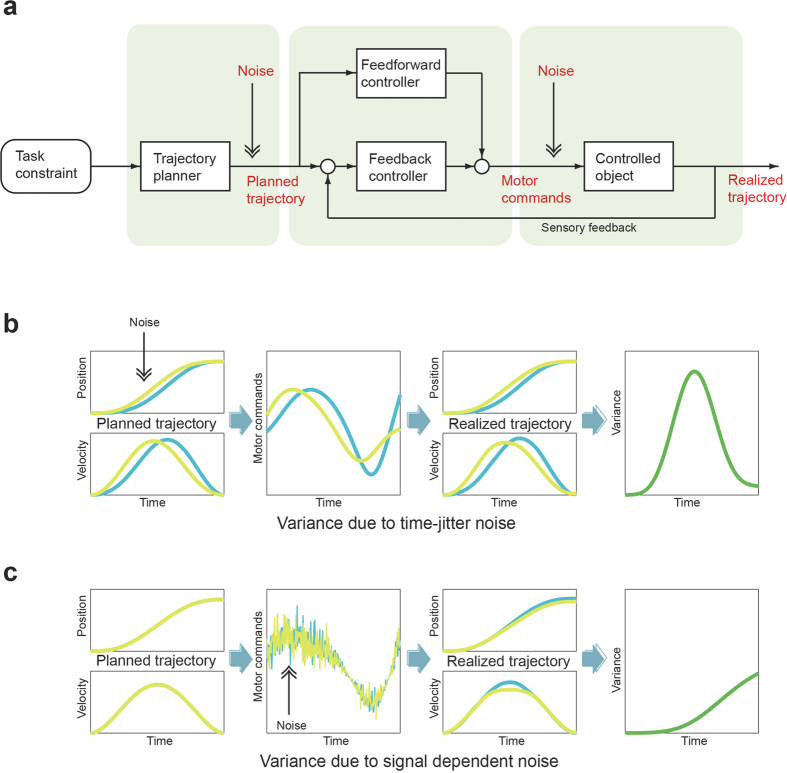
Time-jitter noise versus signal-dependent noise. (**a**) Motor noise can be added both at the level of intermediate representation of the plan (i.e., the output of the planner) and at the level of the motor command (i.e., the output of the controller). (**b**) Effects of time-jitter noise in the desired trajectory on the variance of the produced trajectory. Time-jitter noise is added as a temporal shift (blue) on the position sequence (green). The motor command sequence is then computed from this skewed position sequence, resulting in velocity-dependent increases of variance in the produced trajectory. (**c**) Effects of signal-dependent noise on the variance of the produced trajectory. Because signal-dependent noise is added to the motor command sequence, the effect of the noise is integrated and appears as incremental variance in the produced trajectory.

Because the trajectory variance tends to be more affected by the movement velocity than by the task constraints as seen in the above experiments, we investigated the temporal aspects of the variance in more detail. If a representation of a nominal trajectory exists in the brain for skilled and rapid movements, we hypothesize that noise can be added to that trajectory representation in a manner similar to noise added to the control command. If we assume that the motor command is computed sequentially via a nominal trajectory (Fig. 4a), the motor noise at the level of a nominal trajectory affects the actual trajectory without time delay because it does not yield to the integration effect of dynamics (Fig. 4b). In contrast, motor noise added to the motor command, known as signal dependent noise, plays through dynamics, resulting in an incremental increase of variability in the actual trajectory (Fig. 4c). Therefore, we may be able to distinguish the source of noise by analyzing the temporal aspect of variability in the actual trajectory either at the planning level or at the motor command level.

We propose a novel noise model assuming the time jitter noise in a desired trajectory expressed as a position sequence as a function of time as well as signal-dependent noise. The time jitter noise here means local advance or delay of time in reading out a desired trajectory owing to speed changes of planner dynamics. Employing the proposed model, we separated the variability caused by noise at the planning level from that caused by noise at the motor command level. Specifically, we succeeded in predicting the time course of the trajectory variability *T_Var(t)* during reaching movements using a linear summation of incremental variability coming from the signal-dependent noise and velocity-dependent variability coming from the planning noise (see Methods):


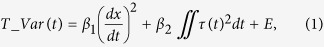


where, the trajectory variance *T_Var(t)* corresponds to the variability of the actual hand position at time *t* from the desired hand position at the same time *t* assuming the representation of a desired trajectory in the brain. Because information of the actual desired trajectory in the experiments with human subjects is not available, we used the mean trajectory as its approximation. In Eq. 1, the first term represents the effect of time-jitter noise on the desired trajectory and the second term represents the effect of signal-dependent noise playing through the dynamics, which is proportional to the double integral of the sum of square of the motor command *τ.* For simplicity, *τ(t)*^*2*^ was approximated by the summation of the square of the shoulder torque and the square of the elbow torque. *E* includes both the spatial noise of the planned trajectory and the modeling error. Thus, the hierarchical schema predicts that the trajectory variance *T_Var(t)* can be reproduced by the linear summation of terms proportional to 1) the square of velocity, 2) the incremental term that is proportional to the integrated motor commands, and 3) the error term.

**Figure 5:**
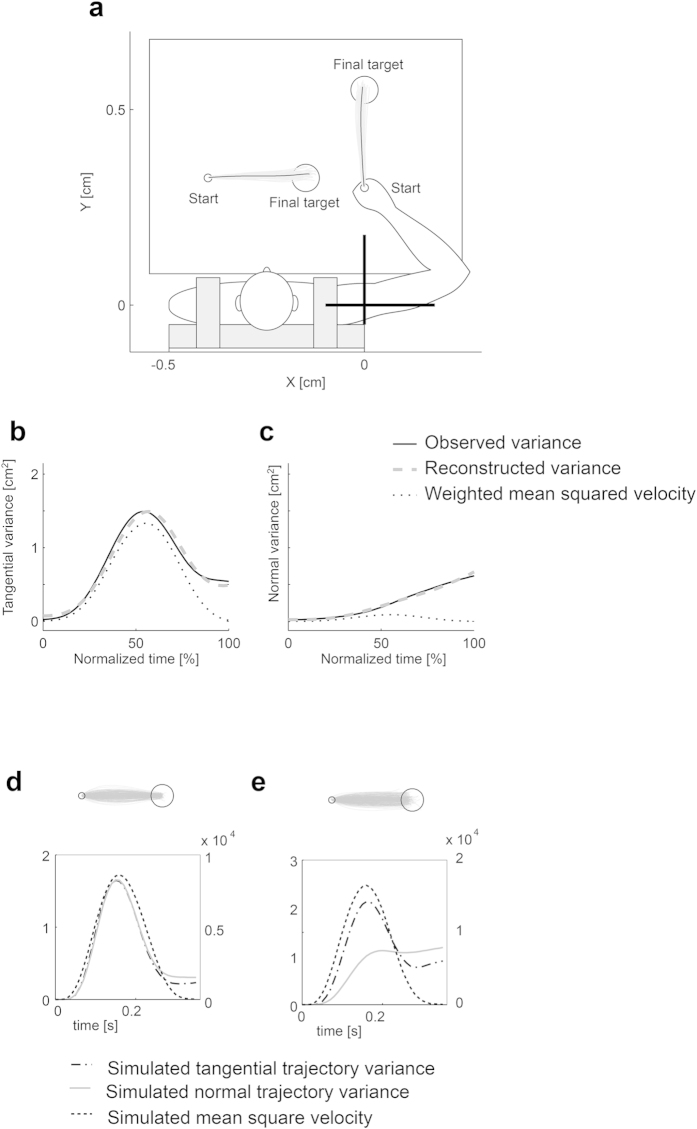
Trajectory variance and model fitting in point-to-point movements and LQG simulation. The participants’ task was to move their hand from the start point (a circle with a radius of 1 cm) to the final target (a circle with a radius of 3.5 cm) within a time limit (350 ± 35 ms). (**a**) Allocations of start and end circles for forward and left-to-right movement tasks, and hand paths of a typical participant. Trajectory variance along the movement direction (tangential variance) (**b**) and trajectory variance perpendicular to the movement direction (normal variance) (**c**) are demonstrated. Solid curves are the observed trajectory variance. The black dotted curves show the mean squared tangential velocity. Gray dashed curves are the trajectory variance reconstructed using Equation 1. (d) LQG simulation results obtained using basic parameter settings (*σ*_*S*_ = 0.4, *σ*_*u*_ = 0.6, *w*_*v*_ = 0.1, *w*_*f*_ = 0.01, *r* = 0.00002). (e) LQG simulation results obtained using biased parameter settings regarding motor noise ([0.9, 0; 0, 0.5], [0, 0.9; −0.5, 0]) and task constraints at the final target position (3.0 for the tangential direction, 0.2 for the normal direction).

The proposed model predicts that the trajectory variance normal to the movement direction has less contribution from the velocity-dependent term because there is a small component of velocity in the normal direction. The trajectory variance tangential to the movement direction, however, should have a significant velocity-dependent term because there is a substantial increase in velocity in the tangential direction. Therefore, *β*_*1*_ should be different when reconstructing either a normal or tangential variance. To confirm this, we tested simple point-to-point reaching movements with nearly straight trajectories (Fig. 5a).

We computed the trajectory variances normal to and tangential to the mean trajectory from 40 movements after enough practice (Methods). For each variance, the parameters *β*_*1*_ and *β*_*2*_ in Eq. 1 were estimated using the least square error method. Figure 5b,c show the time courses of the observed variance (solid curves) and mean square velocity weighted by the parameter *β*_*1*_ (dotted curves) as well as the reconstructed variance (dashed curves) for forward movements. Both the tangential and normal variances were well reconstructed (Table 1). The contribution of the velocity-dependent term was significantly smaller for the normal than for the tangential variance (ANOVA F(3, 8) = 169.25, p < 0.000001 [forward], ANOVA F(3, 8) = 75.50, p < 0.00001 [rightward]). The normal variance was mainly explained by the incremental term relating to the signal-dependent noise while the tangential variance required both incremental and velocity-dependent terms. The LQG simulation (see Methods) predicted a bell-shaped trajectory variance in both the normal and tangential directions (Fig. 5d). To reproduce the observed dissociation between the normal and tangential trajectory variance in the LQG simulation, signal-dependent noise has to be larger in the tangential direction than in the normal direction, and at the same time, the task constraint has to be smaller in the tangential direction than in the normal direction (Fig. 5e).

**Figure 6:**
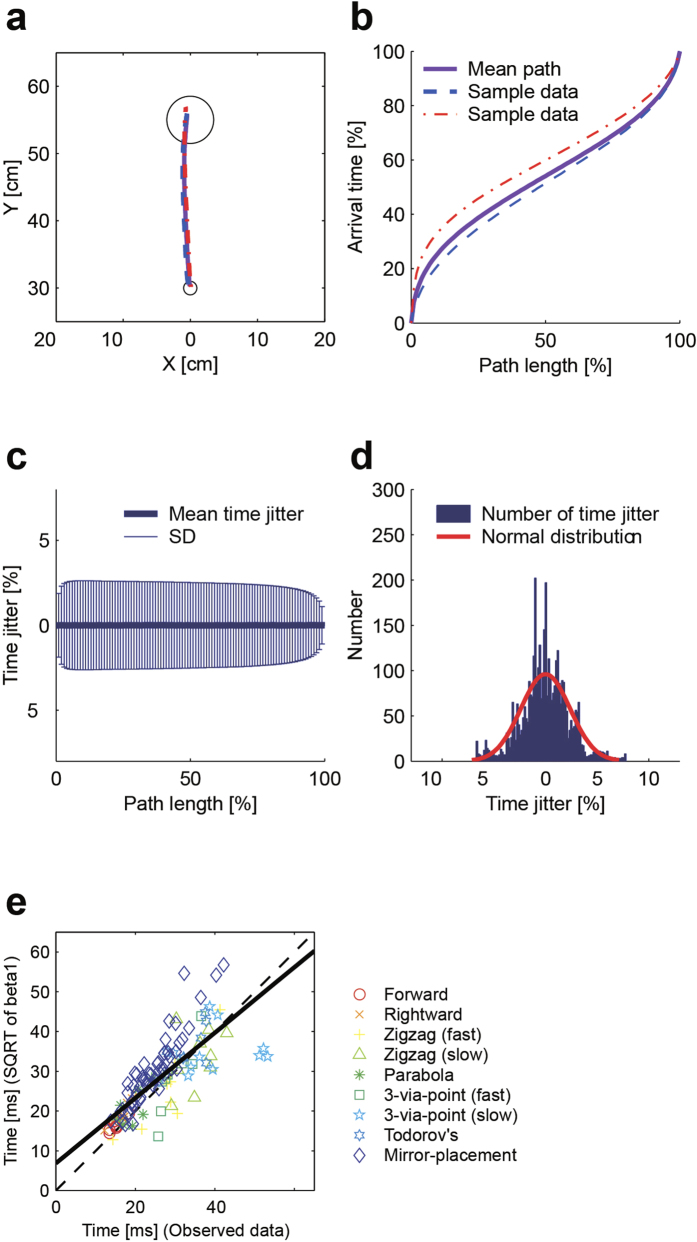
Observed time-jitter noise. The actual distribution of the time-jitter noise was computed from the data as follows. First, the trajectory of each participant was time normalized to remove the effect of the movement duration. This is done because the extension and contraction of the whole movement duration could otherwise possibly be explained by the integrative effect of signal-dependent noise. All trajectories of each participant were then resampled at 100 equally spaced points along the path, and the time at each resampled position on the path was computed for each trajectory. In this way, we removed the effect of global lengthening, shortening, and positional shift of the path, which again can be explained by SDN and/or spatial noise. If we are successful in removing SDN and spatial noise, the resampled position sequence should represent the planned trajectory without spatial noise; i.e., *x*^*k*^_*planned*_*(t)* – *w*^*k*^. Here, the resampled positions that have the same order (percentage) along the path length correspond to the positions at the same time step on the planned trajectory. The mean trajectory of the participant was computed from the resampled trajectories and approximated as the desired trajectory of that task *x*_*desired*_*(t).* Examples of the hand path (**a**) and its arrival time at each position (**b**) were taken from Experiment 2. The difference between the time at each resampled position and the time at the corresponding position of the mean trajectory represents time-jitter noise *δ*(*t*). Assuming that the mean trajectory of one particular task of one participant is the desired trajectory of that task, we computed the mean and standard deviation of time-jitter noise at each position along path (**c**) and its distribution (**d**). (**e**) Square root of *β*_1_ plotted against the standard deviation of the time-jitter noise computed from the data of experiments 1, 2 and 3.

Our noise model assumes that the time at each position on the movement path across many trials has a Gaussian distribution with a mean of zero, and that the standard deviation of this distribution is constant throughout the movement duration (Methods). Independent of the model fittings, we computed the actual distribution of time at each position in the trajectories produced in Experiment 2 to examine the properties of the time-jitter noise. Although the movement paths are nearly identical to each other, the time when the hand reached the same position on the path is slightly different (Fig. 6a,b). For example, the time at the 50% point of one path shown in (b) (dashed curve) was advanced of that of the mean path (solid curve). In contrast, the time at the 50% point of another path (dash-dotted curve) was delayed compared with that of the mean path. The time-jitter noise in the produced trajectories was approximated by the Gaussian distribution with a mean of zero, which is consistent with the model assumption (Fig. 6c,d). From this, we computed the standard deviation of the time-jitter noise for each task, and compared it with the standard deviation of the time-jitter noise predicted from the model (the square root of the parameter *β*_*1*_). The standard deviation of the time-jitter noise computed from the data of Experiments 1, 2 and 3 (see below for the data of Experiment 3) correlated with that estimated from the model fitting (*r* = 0.79, Fig. 6e). The results show that temporal fluctuations in the trajectory had a Gaussian distribution with an approximately constant standard deviation throughout the movement.

### Experiment 3: effect of dynamics on trajectory variance

**Figure 7:**
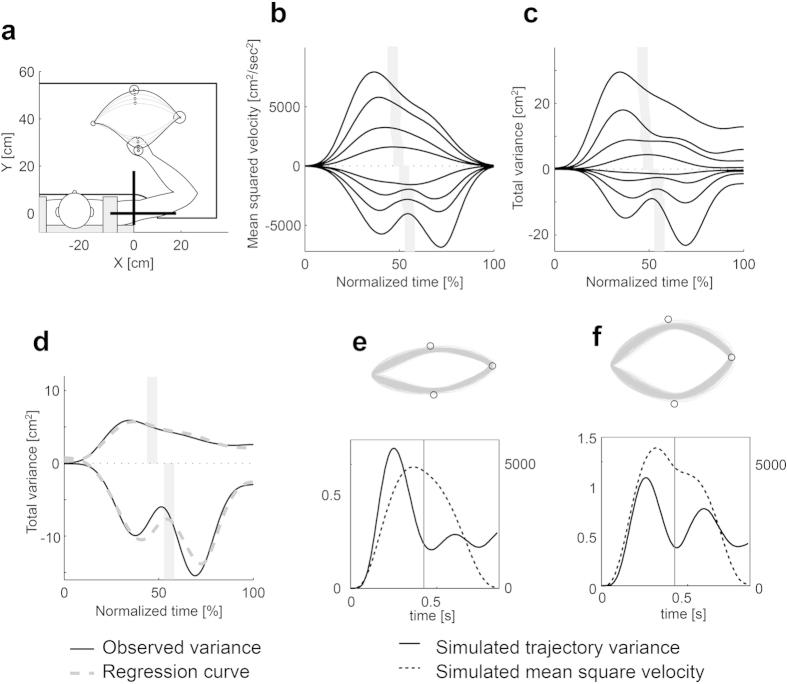
Trajectory variance modulation in mirror-placement tasks and LQG simulation. The participants’ task was to move their arm from the initial position to the final position by passing through a via-point within a time limit (650 ± 50 ms). The start and final target positions were decided according to the participants’ arm joint angles (start point: shoulder 59°, elbow 99°; end point: shoulder 14°, elbow 91°). The different start and target positions were due to the different arm lengths of each participant. (**a**) Allocation of start, via-point and end circles. When the via-point was located farther from the body than the line connecting the start and end points, the mean square velocity profile had a single peak. Conversely, when the via-point was located closer to the body, the profile had two peaks (**b**). The time course of variance had the same features, which can be explained by our model (**c**). The gray areas show the time when the hand passed through the via-point. Magnitudes for each mean squared velocity and variance profile were weighted by the following values for display purposes: (**b**), 1.0, 0.8, 0.5, 0.3, −0.3, −0.5, −0.7 and −1.0; (**c**), 5.0, 4.0, 1.5, 0.5, −0.15, −0.5, −0.55 and −1.50. (**d**) Time course of variances (black solid curves) and variance reproduced by the time-jitter noise model (gray dashed curves) for the most distal and proximal via-point allocation. (**e**,**f**) Results of LQG simulations using parameters *σ*_*S*_ = 0.4, *σ*_*u*_ = 0.7, *w*_*v*_ = 0.2, *w*_*f*_ = 0.02, *r* = 0.002. Upper panels show hand paths. Lower panels show time courses of trajectory variance and mean squared velocity. Vertical lines denote the time specified in the simulation when the hand passes through targets.

It is known that human hand trajectories are affected by the dynamics of the muscle skeletal system of the body^11^. For example, when the location of an intermediate target (via-point) is closer to the body than the horizontal start–end line, the hand velocity tends to exhibit a double-peaked profile^12^. In contrast, it has a single-peaked profile when an intermediate target is located away from the body (Fig. 7a,b). The minimal intervention principle in the Cartesian task space with linear dynamics would predict a symmetric variance modulation with respect to the location of the target around the horizontal line as predicted by the LQG simulation (Fig. 7e,f) where the nonlinear dynamics of the body were not taken into account. However, we observed asymmetric modulation of the trajectory variance similar to the velocity profile (Fig. 7c).

The proposed noise model in Eq. 1 successfully reconstructed the trajectory variance, and the contribution of the velocity-dependent term was significantly positive for all participants (p < 0.00001) and sufficiently large (Fig. 7d, Table 1).

## Discussion

### Characteristics of variance modulation

The present study focused on variance modulation during human reaching movements in both spatial and temporal domains. We demonstrated (1) learning-related reduction of variance modulation and (2) velocity-dependent variance modulation. These results demonstrate convergence towards nominal trajectory after learning and suggest an expression of nominal trajectories in the brain. We identified two different noise sources that could contribute to the time course of movement variability: time-jitter noise in the desired trajectory and signal-dependent noise in the motor commands. This simple model was able to reproduce the variance modulation observed in behavioral experiments. The observed learning-related decrease in task-relevant modulation revealed that movement became more stereotyped, suggesting a reduction in the weight on online optimal feedback in movement. After practice, the feedforward controller around nominal trajectory seems to take over the major part of computation for these movements^19^.

### Explanation of the reduction in variance modulation

It has been argued that the task-relevant modulation of variability (the ‘minimal intervention principle’) is compatible with the optimal feedback control hypothesis but incompatible with the plan-based control hypothesis. However, it is in fact compatible with the plan-based control hypothesis, particularly when considering the process of practice and trajectory optimization. Especially for complex and inexperienced movements, computation of the desired trajectory may not have converged yet at first. Task-relevant modulation observed at the beginning of learning could be partly explained by the exploration of a desired trajectory in addition to the results of optimal feedback control. Suppose that participants encounter a new reaching task with constraints (e.g., the novel allocation of intermediate targets) and practice it several tens of times to meet the constraints. In the plan-based control strategy, the brain first produces a desired trajectory by offline optimization computation. In simulations, a number of iterations in the computation are typically required to determine the optimal trajectory from sub-optimal trajectories, especially when the cost function is complex. It is also probable for a biological system that sufficiently long duration is needed to converge to the optimal trajectory^20^. In such a case, a participant may start reaching with sub-optimally planned trajectories that satisfy the target constraints while exploring the trajectory between targets^21^. Given the intermediate targets as hard constraints, the optimal trajectory planner may try to solve redundancy problems that lie between the targets by optimizing soft constraints such as jerk or energy. Then, at the beginning of practice, the path variance should be smaller at the task constraints and larger between them. After the brain obtains the optimal solution, the trajectory should become a more stereotyped pattern with less modulation around the task constraints. Therefore, the plan-based control hypothesis predicts a practice-related decrease in task-relevant variance modulation.

Optimal feedback control may also be able to explain stereotyped movement after learning if we consider changes of the weights in the criteria in the brain. For example, after learning, the brain might have had smaller weights with respect to the constraint and larger weights on, for example, the effort leading to convergence of the trajectory between targets.

### Feedback control or not

Many attempts have already been made to investigate whether the exclusive use of feedback control is sufficient to explain human arm movements. Theoretical work has demonstrated that rapid and coordinated arm movements cannot be executed solely under feedback control because biological feedback loops are slow and have small gains^22^. Miall *et al.* suggested an idea of combining the forward model and feedback controller as an inverse model to compensate time delay^23^. However, Mehta and Schaal showed that this strategy could not stabilize an unstable system^24^. Therefore, the exclusive use of a feedback control law does not seem to be practically useful in a biological system with time delay, whereas a feedforward impedance controller can stabilize unstable dynamics by learning appropriate impedance^25^. In addition, as illustrated in Experiment 3, optimal feedback control with linear dynamics was not able to predict asymmetric trajectory and variance profiles, and it is non-trivial to derive a general optimal feedback controller for nonlinear plant dynamics^26^. To our knowledge, there is still no effective demonstration of dealing with nonlinear dynamics or unstable dynamics through the exclusive use of optimal feedback control in the literature on biological motor control. The iterative linear quadratic Gaussian (iLQG) scheme, as one solution for the nonlinear dynamics, includes both feedforward commands and local optimal feedback around the optimal trajectory^27^. In this case, the feedforward motor command is computed without hierarchical computation, as a result of optimization using an internal model.

Several recent studies reported physiological and behavioral results supporting optimal feedback control^28^,^29^,^30^,^31^,^32^. For example, representations of MI neurons do not always remain constant across behavioral contexts but change their sensitivity according to the task^33^. There exists a flexible and sophisticated long latency reflex that is similar to the later voluntary response^34^ and has an internal model of limb dynamics^35^. Diedrichsen successfully demonstrated that both feedback control and the adaptation of two hands change optimally according to the current bimanual task requirements^36^.

The feedback controller itself is consistent with the desired trajectory hypothesis. Some recent models have progressively incorporated the feedback controller into the feedforward controller with a trajectory planner^37^,^38^. These models can deal with interactive movements with objects as well as target shifts during the movements. Much evidence suggests the existence of sophisticated long-latency feedback, and it is possible that such high-level feedback systems are dedicated to an optimal feedback control law. However, it is still unclear whether the feedback gain is in fact effectively modified online so that it can optimize the performance. The gain may be sophisticated but sub-optimal^39^ and it may be already preplanned and executed in a feedforward manner^25^. By controlling stiffness (i.e., the feedback gain using a feedforward mechanism), the effect of signal-dependent noise can be reduced without complicated online computation of feedback gains^40^. This schema (i.e., desired trajectories combined with feedforward impedance control) allows us to explain the decrease in variance at task constraints^5^,^41^. The variance at the targets can be decreased because the trajectories are corrected towards the desired trajectory with a preplanned gain that is higher than that at midpoints. Thus, even if the path variance were to decrease near via-points, we could not conclude that the motor control system employed the optimal feedback control law. Further investigations will be performed to demonstrate the existence of real-time and optimal modulation of feedback gain.

There is also recent physiological evidence for feedforward control. Subcortical structures can contribute to prepared motor responses in humans through reticulospinal tract, although its fundamental role is limited to coordinated movement of the whole hand, rather than dexterous individual finger movements^42^,^43^. Subcortical structure of rats, without motor cortex, has recently been shown to execute skilled, but not dexterous motor tasks, although motor cortex was necessary during the process of learning the tasks^44^. These results demonstrate that subcortical structures may play an important role in feedforward control of fundamental motor repertories. Better understanding of these interactions between cortical and subcortical structures may help elucidate conditions and tasks for which feedforward and feedback control strategies are most relevant.

### Noise in the nominal trajectory

Previously, Gordon *et al.* measured variability at the end of a movement to determine the nature and origin of the coordinate system in which the movements were planned^45^. McIntyre *et al.* further examined different types of errors to identify properties of the internal representation and coordinate transformations in the brain^46^. Churchland *et al.* showed that at least 30% of behavioral variability can be accounted for by the variability of preparatory neural activity in the dorsal premotor cortex^20^. While these studies assume that observed variability mainly or partly arises in the planning process, van Beers *et al.* successfully explained the variability at the end of movement by noise in movement execution (i.e., noise in the motor commands (signal-dependent noise^47^)) rather than by noise in the desired trajectories^48^.

In our noise model, we hypothesized that time-jitter noise exists at the planning level but not at the motor command level. If the time series of motor commands were temporally stored before being issued, or expressed in such a way as a table lookup method, time-jitter noise could also appear when the stored motor commands were read out at each moment. However, because the effect of noise on the motor commands plays through the integrating effect of arm dynamics, it will also result in the incremental increase of path variance, mainly expressed as the global extension and contraction of movement duration and overshoot/undershoot at the end of the movement. Therefore, time jitter noise on the motor command will not correlate with velocity and should be removed by time normalization and/or be included in the second incremental term of Eq. 1.

### Combination of the preplanned trajectory and local optimal feedback

Our overall results suggest that online feedback control around task constraints is not the exclusive strategy of motor control and, at least for rapid skilled movements, desired trajectories are planned in the brain. The most possible and feasible solution would be the combination of a desired trajectory and local optimal feedback control. Using a preplanned trajectory, the brain can solve complex problems with nonlinear dynamics before starting the movement through the learned internal model. The brain makes the best use of offline computation to reduce the cost of online computation while online computation concentrates on further fine tuning and dealing with unpredictable perturbations using redundant degrees of freedom^19^,^32^. The principles of biological motor control will be further investigated in future work.

## Methods

### Participants and experimental setting

Twenty-four male and one female participants, aged 21–32 years, who were right-handed except for one, participated in at least one of the three experiments. Seventeen participated in the multi-via-point tasks (Exp. 1), nine in the point-to-point movement tasks (Exp. 2), and seven in the mirror-placement single-via-point tasks (Exp. 3). Seven participated in both Experiments 1 and 3, and another four participated in both Experiments 1 and 2. All experiments were conducted in accordance with the principles and the guidelines in the Declaration of Helsinki and were approved by the ATR Human Subject Review Committee. The participants provided informed consent before participation.

Participants were seated on a chair and their shoulders fixed to the back of the chair with a harness. The height of the table was adjusted to lift the participant’s arm to shoulder level. The participant’s right wrist was braced so that movement was constrained to allow only two degrees of freedom of the elbow and shoulder. To reduce friction between the arm and table, the arm was attached to a board that was levitated above the table by an air sled. The participants performed all tasks with their right hand. An OPTOTRAK 3020 device (Northern Digital Inc., Canada) was used to measure the position of a marker placed on the end of a 9-cm vertical bar that was grasped by the participant. The marker position was sampled at 500 Hz (Experiment 2) and 400 Hz (Experiment 1 and 3) and projected as a cross mark on a high-resolution monitor placed in front of the participants to represent the current hand position. The participants performed the experiment while looking only at the monitor. The room was darkened to eliminate visual information, and the participant wore noise-canceling headphones (Bose QuietComfort Acoustic Noise Canceling headphones, Bose Corporation, USA) to eliminate auditory noise and to allow him/her to concentrate on the individual experiment. We used a beep sound to indicate the beginning and the end of the movement task.

#### Experiment 1: multiple target tasks

Nine participants performed Todorov’s task (three intermediate target conditions of Experiment 1 in ref. 1), the slow zigzag task, and the slow three via-point task while the other eight participants performed the parabola task, the fast zigzag task, and the fast three via-point task (Fig. 1). The participants performed each task until there were 50 successful trials. One participant could not complete the fast three-via-point task, which was excluded from the analysis (while other tasks of this participant were included in the analysis). The average numbers of trials were 345 ± 85 for Todorov’s task, 132 ± 31 for the parabola task, 109 ± 24 and 106 ± 20 for the fast and slow zigzag tasks, and 133 ± 34 and 148 ± 35 for the fast and slow three-via-point tasks. The peak velocity after learning (average of the last 20% of the total trials) averaged across participants was 43.67 ± 4.66 cm/s for Todorov’s task, 57.71 ± 9.26 cm/s for the parabola task, 49.87 ± 7.66 and 30.38 ± 2.01 cm/s for the fast and slow zigzag tasks, and 61.51 ± 13.89 and 36.65 ± 3.04 cm/s for the fast and slow three via-point tasks respectively.

#### Experiment 2: point-to-point reaching task

Experiment 2 consists of two sessions of point-to-point reaching tasks along different directions. In one session, participants performed movements in the forward direction, and in the other session, they moved in the rightward direction (Fig. 5). Movements ending in the target circle within the specified duration were regarded as successful trials. The participants were randomly placed into one of the two groups. On the first day, one group practiced forward movements until they reached 50 successful trials followed by rightward movements. The second group started with rightward movements followed by forward movements. On the second day, the first group performed forward movements until they reached 40 successful trials followed by rightward movements. The second group started with rightward movements. The average success rates on the second day were 78.72 ± 12.37% for forward movements and 79.65 ± 5.52% for rightward movements.

#### Experiment 3: single via-point tasks (mirror-placement tasks)

Eight intermediate targets were selected and equally arranged on the perpendicular bisector of the start–goal straight line (Fig. 7). The combination of the start point, end point and via point was regarded as a set. One of the eight sets was randomly presented for each task. First, the participants performed eight sets of 20 tasks for a total of 160 tasks (training). Second, the participants were trained for four via-points that were selected on the basis of the failure rate. Forty trials were performed for each via-point, amounting to 160 tasks (training). Finally, the participants performed the same task as in the first step. Equation 1 was applied to the trajectories in the last step.

### Data analysis

Position data were digitally filtered using a third-order Butterworth low-pass filter with a cutoff frequency of 12 Hz. The velocity was computed by applying a three-point derivative of the measured position data. The start and end points of each movement were determined using a curvature threshold of 100 m^−1^ 49. Trials that did not stop within the final target circle and trials whose movement duration or path length was more than 3 standard deviations from the mean were excluded from further analyses.

For the purpose of examining the task-relevant variance, we applied the methods used by Todorov and Jordan^1^ where data were normalized on the basis of path length. This method removes the temporal effects. First, all trajectories for one participant and condition were resampled at 100 equally spaced points along the path. Second, the average trajectory was computed from the resampled data. Third, for each average point, the nearest point from each trial was found and the path variance was calculated from that point. To avoid artifacts of realignment, 5% of the path at each end was eliminated from the analysis.

The modulation index was computed from the path variance as follows.


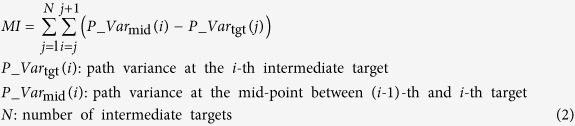


here, *P_Var*_*tgt*_(*i*) denotes the variance at the *i*-th target and *P_Var*_*mid*_(*i*) denotes the variance at the mid-point between the (*i*-1)-th and *i*-th targets. *N* denotes the number of intermediate targets. The variance at a certain target was defined as the minimum value observed within 5% in front of and behind the target along the path. The variance at a certain mid-point was defined as the maximum value observed within 5% in front of and behind the mid-point along the path.

For the purpose of examining the temporal characteristics of the variance and the effect of time-jitter noise, the trajectory variance of each participant was computed for a set of trajectories as follows. First, the data were resampled between the start and end times so that the duration was evenly divided into 100 pieces to remove the effect of movement duration (time normalization). Therefore, each trajectory has 100 data points with different sampling intervals depending on the movement duration. Second, the resampled position was ensemble averaged to compute the mean position for each 100 time steps. Trajectories whose movement duration and path length were within 2 standard deviations of the mean were included in the regression analysis.

We assume that a trajectory pattern (i.e., a position sequence as a function of time) of the *k*-th trial 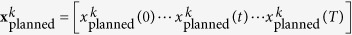 is generated by the trajectory planner. The position at each time step *t* of this planned trajectory pattern is expressed as


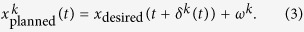


Here *x*_desired_ denotes the desired trajectory of that task; i.e., a position sequence as a function of time in the absence of noise. Each planned trajectory spatiotemporally deviates from the desired trajectory because of the noise in planner dynamics and computational limitations. *δ*(*t*) represents the local advance or delay of time (time jitter noise) and *ω*(*t*) represents spatial noise.

This time series of the planned position 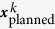 is read out at each moment. The motor command at each time step is computed using an inverse model of the controlled object such as the arm. For notational simplicity, we use *G* to denote the dynamics of the controlled object (i.e., the arm) and *G*^−1^ to denote its inverse model. Assuming that signal-dependent noise 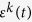 is added to motor commands when they are issued to the controlled object, the actual produced trajectory 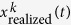 can be approximately expressed as


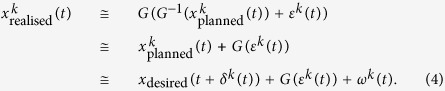


Here, *δ*(*t*), *G*(*ε*(*t*)), *ω*(*t*) are assumed to have a normal distribution with zero mean and standard deviation *a*(*t*), *b*(*t*), *c*(*t*), respectively (see below). The produced trajectory is approximated by the Taylor expansion to the second degree:


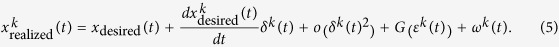


Assuming that 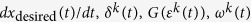 are mutually uncorrelated, the mean and variance of the produced trajectory are


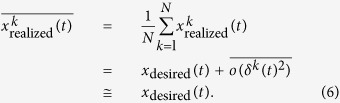



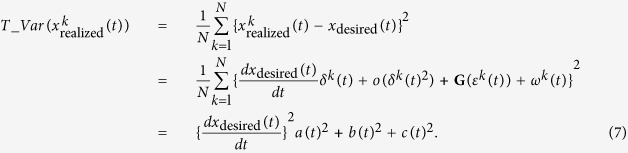


Because *b*(*t*) is the effect of signal-dependent noise playing through the dynamics, it should be proportional to the double integral of the sum of the square of the motor command *τ*. Here we used the joint torque to approximately estimate the magnitude of the motor commands. *a*(*t*) and *c*(*t*) are the time jitter and spatial noise of the planned trajectory, respectively, both of which are independent of the signal and dynamics. Assuming that *a*(*t*) is constant throughout the movement, the trajectory variance is modeled as in Eq. 1.

The *total trajectory variance is* defined as the sum of the *x* and *y* variances:


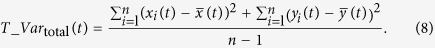


The *trajectory variances in tangential* and *normal directions* are computed from the variance in the direction tangential and normal to the mean trajectory respectively:


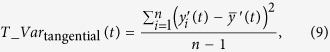



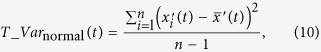


where *y*′ and *x*′ denote the tangential and normal components of position with respect to the mean trajectory, respectively.

For the purpose of regression analysis of Eq. 1, dynamic torques were calculated using the dynamics equations of a two-link arm model and the position data and link parameters estimated from the link length for each participant (with the data of an adult male arm measured with a three-dimensional scanner to provide a standard). The mass of links was adjusted for each participant by making the standard value proportional to the link length of the participant. The moment of inertia of the links was adjusted by making the standard value proportional to the third power of the link length of the participant. Viscosity coefficients were estimated from the absolute average torque for each movement using the equation (6) in ref. 3.

#### Optimal feedback control simulation

In the comparative LQG simulations for the reaching tasks in Experiments, the LQG formulation described in the supplementary information in ref. 1 was used (refer to Section 2 for details). The same parameters for the point mass (*m* = 1 kg), the time constant for the filters (*τ* = 40 ms) and the sampling time for discretization (Δ*t* = 10 ms) were used as in ref. 1. The sensory noise parameter *σ*_*S*_ and the control noise parameter *σ*_*u*_ were adjusted so that simulated variability approximately matched the experimental data. The weight parameters in the cost defining the relative importance of stopping for the velocity and force terms respectively (*w*_*v*_, *w*_*f*_) were also adjusted for the experimental data. The weight for the effort penalty *r* was adjusted for each task (either *r* = 0.002 or *r* = 0.00002 similarly as in ref. 1). See the figure legend for the parameter settings for each simulation.

## Additional Information

**How to cite this article**: Osu, R. *et al.* Practice reduces task relevant variance modulation and forms nominal trajectory. *Sci. Rep.* **5**, 17659; doi: 10.1038/srep17659 (2015).

## Figures and Tables

**Table 1:** Results of multiple regression.

Task	R^2^	β_1_	β_2_
*Todorov*	0.61 ± 0.15	0.82 ± 0.12	0.40 ± 0.18
*Parabola*	0.53 ± 0.23	0.78 ± 0.17	0.47 ± 0.24
*Zigzag (fast)*	0.53 ± 0.21	0.70 ± 0.17	0.05 ± 0.24
*Zigzag (slow)*	0.54 ± 0.18	0.70 ± 0.09	0.10 ± 0.24
*Three via-points (fast)*	0.54 ± 0.26	0.70 ± 0.24	0.33 ± 0.15
*Three via-points (slow)*	0.41 ± 0.22	0.60 ± 0.19	0.26 ± 0.25
*Forward normal*	0.97 ± 0.04	0.19 ± 0.13	1.00 ± 0.03
*tangential*	0.97 ± 0.02	0.99 ± 0.03	0.38 ± 0.10
*Rightward normal*	0.98 ± 0.02	0.20 ± 0.21	0.96 ± 0.06
*tangential*	0.98 ± 0.02	0.95 ± 0.05	0.36 ± 0.11
*Mirror placement*	0.81 ± 0.10	0.93 ± 0.03	0.34 ± 0.10

standardized coefficients ± SD.
